# RNA-sequencing analysis of fungi-induced transcripts from the bamboo wireworm *Melanotus cribricollis* (Coleoptera: Elateridae) larvae

**DOI:** 10.1371/journal.pone.0191187

**Published:** 2018-01-16

**Authors:** Bi-huan Ye, Ya-bo Zhang, Jin-ping Shu, Hong Wu, Hao-jie Wang

**Affiliations:** Research Institute of Subtropical Forestry, Chinese Academy of Forestry, Hangzhou, China; Uppsala Universitet, SWEDEN

## Abstract

Larvae of *Melanotus cribricollis*, feed on bamboo shoots and roots, causing serious damage to bamboo in Southern China. However, there is currently no effective control measure to limit the population of this underground pest. Previously, a new entomopathogenic fungal strain isolated from *M*. *cribricollis* larvae cadavers named *Metarhizium pingshaense* WP08 showed high pathogenic efficacy indoors, indicated that the fungus could be used as a bio-control measure. So far, the genetic backgrounds of both *M*. *cribricollis* and *M*. *pingshaense* WP08 were blank. Here, we analyzed the whole transcriptome of *M*. *cribricollis* larvae, infected with *M*. *pingshaense* WP08 or not, using high-throughput next generation sequencing technology. In addition, the transcriptome sequencing of *M*. *pingshaense* WP08 was also performed for data separation of those two non-model species. The reliability of the RNA-Seq data was also validated through qRT-PCR experiment. The *de novo* assembly, functional annotation, sequence comparison of four insect species, and analysis of DEGs, enriched pathways, GO terms and immune related candidate genes were operated. The results indicated that, multiple defense mechanisms of *M*. *cribricollis* larvae are initiated to protect against the more serious negative effects caused by fungal infection. To our knowledge, this was the first report of transcriptome analysis of *Melanotus* spp. infected with a fungus, and it could provide insights to further explore insect–fungi interaction mechanisms.

## Introduction

Larvae of the beetle superfamily, Elateroidea, are agricultural pests that feed on germinating seeds, seedlings, tubers, root tubers, etc. and are distributed worldwide[1–3] The bamboo wireworm (*Melanotus cribricollis*; Coleoptera: Elateridae) pose danger to bamboos, especially the *Phyllostachys* spp.[4]. Larvae not only feed on bamboo shoots but also injure root systems, reducing bamboo shoot outputs and commodity value, thus, severely affecting the sustainable regeneration of bamboo forests. Recently, bamboo wireworm larvae have become rampant in southern China due to the transformation of tillage system and cultivation of a single fast-growing bamboo species [5]. Larvae of *M*. *cribricollis* are predominant in southern China and account to about 3 wireworms per kilogram of cultivated bamboo[6] and even 19 wireworms have been found in one bamboo shoot[5,7]. Current measures to control this pest include the use of conventional insecticides, such as carbofuran and clothianidin. However, due to pesticide resistance, environment pollution, food safety issues and various other negative impacts, alternative pest control strategies are urgently needed. Entomopathogenic fungi are naturally available infectious agents that can be used as complementary biocontrol measures. *Metarhizium* spp. are broad-spectrum fungi, which have been demonstrated to effectively control more than 200 pest populations [8] including the underground wireworms, *Agriotes obscurus* [9–10], *Limonius californicus* and *Hypnoidus bicolor* [2]. Previously, a new fungal strain *M*. *pingshaense* WP08 with high toxicity, was isolated from cadavers of *M*. *cribricollis* larvae in the bamboo forestry, which will probably be an alternative tactic for the effective control of this soil dwelling pest [11].

With the rapid development of sequencing technology, an increasing number of insects have been sequenced, but the bamboo pest *M*. *cribricollis* and the new entomopathogenic fungi *M*. *pingshaense* WP08 strain. Therefore, the Illumina High-Seq technology was employed here for both of them, and several key BP GO terms, pathways and immune related genes of *M*. *cribricollis* larvae were identified. This was the first report to reveal the genetic background of these two organisms, which will be meaningful to unravel the basic mechanisms of insect–fungi interaction, and might lead to alternative biological pest control strategies to some extent.

## Materials and methods

### Insect and fungi

The *M*. *cribricollis* larvae tested were maintained in climate chambers at 25±1°C, 10±1% soil humidity in the dark. The entomopathogenic fungus, *M*. *pingshaense* WP08, was cultured on PPDA medium in climate chambers at 25±1°C and 85±10% relative humidity. Conidia harvested after three weeks were suspended in 0.05% Tween 80.

### Biological assay of *M*. *cribricollis* larvae infected with *M*. *pingshaense* WP08

Larvae with similar size were selected and raised in soil treated with or without conidial suspension (from above). The designed concentration of the treatment group was 3×10^8^ conidia/g soil, as the control group, soil was treated with same volume of 0.05% Tween 80. The other raised conditions (temperature, soil humidity and dark circumstance) were the same as mentioned above. The larval mortality was recorded regularly (1–2 d), and 75 larvae were tested from each group with three replications.

### Sampling and RNA preparation

Five larvae were collected from both CK and infected groups after 1 d, 3 d, 4 d, 5 d and 7 d post-treatment and rinsed with sterile water. Conidia of *M*. *pingshaense* WP08 were also collected from the sterile colony for RNA extraction after cultivation for about three weeks. The samples were immediately frozen in liquid nitrogen. RNA was then extracted using the Quick RNA isolation Kit (Huayueyang Biotech Co., Ltd., Beijing, China). The extracted RNA was treated with RNase-free DNaseI (TaKaRa Biotech Co., Ltd., Dalian, China) to remove residual DNA. RNA integrity was checked on a 1.2% agarose gel by electrophoresis, and RNA concentration was estimated using an Agilent 2100 Bioanalyzer (Agilent Technologies, Inc., Santa Clara, CA, USA).

### Construction of cDNA library and Illumina sequencing

High-quality RNA samples from *M*. *cribricollis* larvae were sent to Biomarker Technologies Corporation (Beijing, China) for cDNA libraries construction and sequencing. Briefly, mRNA was purified by the interaction between the poly (A) tails and magnetic oligo (dT) beads. RNA-Seq libraries were generated using the TruSeq RNA Sample Prep Kit (Illumina, San Diego, CA) with multiplex primers, according to the manufacturer’s protocol. The cDNA library was constructed with an average insert size of 200bp, with non-stranded library preparation. The cDNA was purified using a QiaQuick PCR extraction kit (Qiagen, Inc., Hilden, Germany) and the short cDNA fragments were subjected to end repair, adapter ligation, and agarose gel electrophoresis. Then, fragments of 300-400bp were selected as templates for PCR amplification. Sequencing was performed via a paired-end 125 cycle rapid run, on 2 lanes of the Illumina HiSeq2500, generating approximate 800 million pairs of reads.

### Data filtering and *de novo* assembly

High-quality clean reads were obtained by removing the adaptor sequences, duplicated sequences, ambiguous reads (‘N’), and low-quality reads. Uninfected and infected transcriptomes were separately assembled *de novo* using Trinity (http://trinityrnaseq.sourceforge.net/). In brief, clean reads with an overlap length of 35 bp were initially combined to form long fragments without N (named contigs). Related contigs were clustered using the TGICL software [12] to yield unigenes (without N) that cannot be extended on either end, and redundancies were removed to acquire non-redundant unigenes (-l = 35; identify = 90%). Since both *M*. *cribricollis* larvae and *M*. *pingshaense* WP08 are non-model species, *de novo* assembly of those two control groups were performed, separately.

### Functional annotation of the assembled unigenes

The unigene sequences from both the control and infected groups were BLASTx against the NCBI Nt and Nr, KEGG, GO, COG, Swiss-Prot, and TrEMBL databases (E-value ≤1E-5) to retrieve functional annotations of predicted proteins based on amino acids sequence identity. High-priority databases, followed by Nr, Swiss-Prot, and KEGG, were selected to determine the direction of the unigene sequences. The best alignments were used to predict the coding regions of unigenes, and the coding sequences (CDSs) were translated into amino acid sequences using the standard codon table. The ESTScan software [13] was used to determine sequence direction of unigenes that could not be aligned to any of the above databases. GO terms were assigned to each sequence annotated by BLASTx against the Nr database using the Blast2GO program with the E-value threshold of 1E-5 for further functional categorization. The WEGO software [14] was used to plot the distribution of the GO functional classification of the unigenes. The unigene sequences were also aligned to the COG database to predict and classify possible functions and to the KEGG pathway annotations to analyze inner-cell metabolic pathways and the related gene function.

### Comparative analysis of gene families

The translated protein sequences of *M*. *cribricollis* larvae transcriptome were compared with other three draft genomes of *Drosophila melanogaster* (Diptera), *Dendroctonus ponderosae* and *Tribolium castaneum* (Coleoptera), to obtain amino acids sequence identity across species. Homologous genes were grouped together in the same cluster using the OrthoVenn program provided by the USDA (http://probes.pw.usda.gov/OrthoVenn/start.php).

### Analysis of the functional enrichment of DEGs

The FPKM (Fragments Per Kilobase of transcript per Million mapped reads) was used to eliminate the influence of different gene lengths and sequencing levels in the calculation of gene expression. Therefore, FPKM values were directly used to compare gene expression differences between different samples. The DESeq package was used to obtain the “base mean” value for identifying differently expressed genes (DEGs). FDR ≤0.05 and the absolute value of log2 ratio ≥1 were set as thresholds to determine the significance of gene expression difference between two samples.

Comparison of up-/down-regulated DEG in the control group vs. five treatment libraries was completed. Comparisons of the DEG number in control vs. 7 d with other four groups (control vs. 1 d, control vs. 3 d, control vs. 4 d and control vs. 5 d) was performed, and a Venn diagram was established. To determine which pathways were influenced by fungal infection, DEGs were mapped to KEGG pathways and enrichment analysis (p≤0.05) was conducted. As the “Biological Process (BP)” GO term could reflect various biological processes in vivo, BP GO term enrichment (p≤0.05) was also considered.

### Quantitative RT-PCR

Total RNA was extracted from each sample and the concentration was measured using NanoDrop 2000c Spectrophotometer (Thermo Scientific). Less than 1 μg of RNA was used as a template in reverse transcription to produce cDNA by using the PrimeScript^™^ RT reagent Kit with gDNA Eraser (Taraka Biotechnology, Dalian). Then, SYBR^®^ Premix Ex Taq ^™^II (Tli RNaseH Plus) (Taraka Biotechnology, Dalian) was used for qRT-PCR reactions containing 1 μl cDNA and 0.5 μl of each primer (10 μM) in 20 μl reaction volumes. Three reactions were maintained for each sample. Thermal cycling was performed at 95°C for 30s, followed by 40 cycles at 95°C for 5s, and 60°C for 34°C. Melting curve analysis was performed from 60°C to 95°C. Several house-keeping genes, β-actin, GAPDH, SYN1, SYN6, RPS3, RPS18, RPS13α and α-tubulin, were tested as internal controls for normalization and finally α-tubulin gene was selected. Sequences of the primers were obtained from the closely related *T*. *castaneum* [15]. Primers were designed by IDT (http://sg.idtdna.com/scitools/Applications/RealTimePCR).

### Selection and analysis of immune related unigenes

According to the analysis results of DEGs and immune-related BP GO term, unigenes that were differentially expressed or participated in several important immune-response BP GO terms were selected, then the “Swiss-Prot Hit” description was acquired by comparing the encoding protein sequences of the above unigenes with the other three insect species. Finally, some previously reported immune-related unigenes were selected and discussed. The average FPKM values and the average 2-ΔΔCT values obtained from qRT-PCR were analyzed after data normalization ($\text{zscore}=\frac{x-\bar{x}}{\sqrt{\frac{{\left(x-\bar{x}\right)}^{2}}{n}}}$). Heat-map was constructed using Heml 1.0: Heatmap Illustrator software [16]. Phylogenetic analysis of GNBP was conducted using MEGA version 6 [17].

## Results

### Mortality rate of infected *M*. *cribricollis*

**Fig 1:**
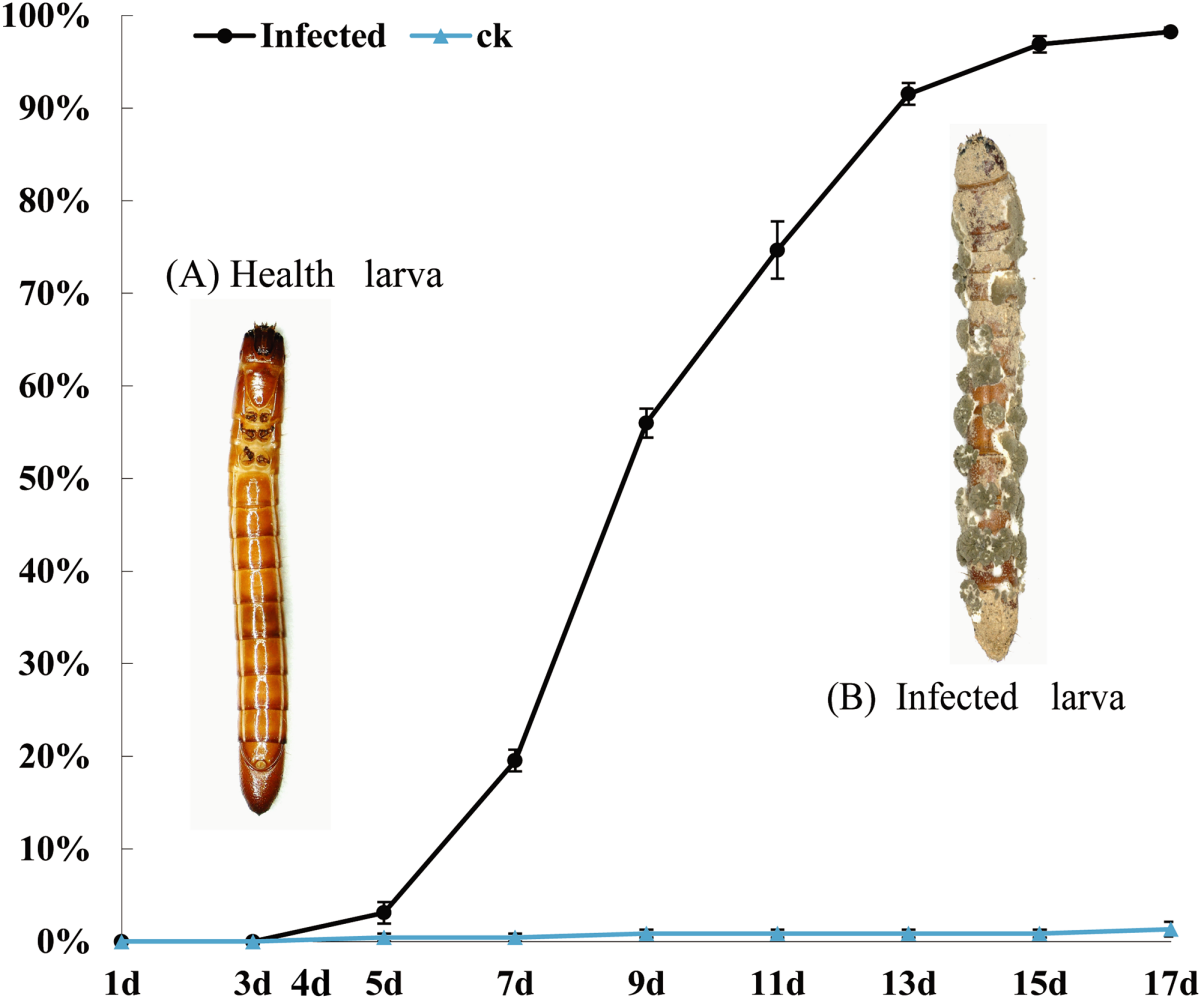
Mortality rate of *M*. *cribricollis* larvae infected with *M*. *pingshaense* WP08. Error bars: SE of the mean. The red marked circle indicates five sample points.

After conidia treatment, the mortality was initially observed on 3 d, and increased markedly from 5 d to 7 d (20%); thereafter, mass mortality occurred, and all experimental larvae (100%) died at 17 d (Fig 1).

### Sequence and *de novo* assembly

In total, the transcriptome sequencing yielded 776,670,786 reads with a GC content of each sample 40–42% and a Q30 value between 95–97% (S1 Table). A close agreement between the three technical replicates was confirmed by the Pearson correlation coefficients. Therefore, the data from parallel libraries were combined for further analyses (S2 Table). The assembly of *M*. *cribricollis* transcriptome resulted in 10,727,315 contigs, 126,031 transcripts and 63,973 unigenes with N50 lengths of 46 bp, 2382 bp and 1670 bp, respectively. The average unigene size larger than 1000 bp was 12,356 (19.32%) (S3 Table). Furthermore, *de novo* assembly of *M*. *pingshaense* WP08 was also accomplished, which resulted in 28,107 unigenes with a N50 length of 2547 bp, and more than 32% unigenes were found to be larger than 1000 bp (S4 Table). We found that more than 80% reads from each larvae library mapped to the *de novo* assembly of *M*. *cribricollis*. The mapped ratio of CK-Metar was also 81.35%; however, the ratio of *M*. *pingshaense* WP08 from infected larvae mapped to the *de novo* assembly of *M*. *pingshaense* WP08 was only 7–10% (Table 1).

**Table 1:** Summary of mapped ratio.

Library		Mapped to *Melanotus*		Mapped to *Metarhizium*	
	Clean Reads	Mapped Reads	Mapped Ratio	Mapped Reads	Mapped Ratio
**1 d**	50,219,120	42,034,448	83.69%	4,579,280	9.05%
**3 d**	41,198,686	34,066,038	82.83%	3,559,646	8.59%
**4 d**	46,583,497	38,571,587	82.71%	3,744,084	8.04%
**5 d**	45,488,455	37,785,893	83.08%	3,433,629	7.57%
**7 d**	37,236,430	30,597,124	82.16%	3,005,193	8.11%
**CK-Melan**	38,164,074	32,012,743	83.81%	NA ^a^	NA
**CK-Metar**	44,623,174	NA	NA	36,437,642	81.35%

^a^ NA indicates not applicable.

### Functional annotation of the assembled unigenes

Results showed that a total of 25,210 (39.41%) unigenes were annotated based on the NCBI-Nr, Swiss-Prot, KEGG, COG, KOG, GO and Pfam databases, which meant 60.59% unigenes had no homologous genes/proteins. Among the annotated unigenes, a vast majority (97.33%) were annotated against the NCBI-Nr database, and more than half were long unigenes (length ≥1000) (S5 Table).

### Comparative analysis of gene families yields common clusters in related insect species

**Fig 2:**
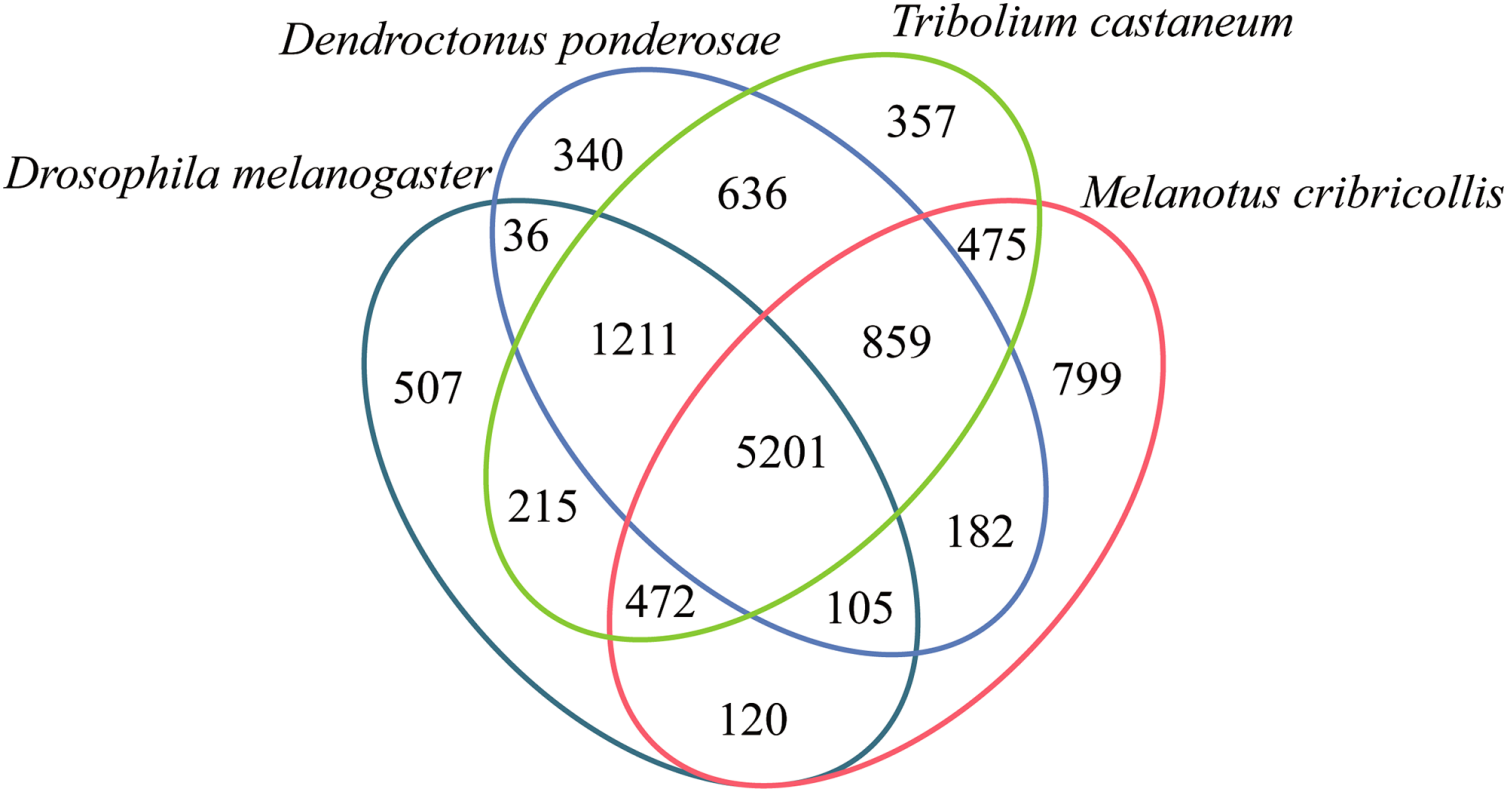
Comparative analysis of four gene families. Comparative summary of predicted amino acids sequences of *M*. *cribricollis* unigenes with protein sequences from three insect species. Amino acid sequence comparison was performed using BLASTx with predicted protein sequences from *D*. *melanogaster* (Diptera), *T*. *castaneum* (Coleoptera) and *D*. *ponderosae* (Coleoptera).

The predicted amino acid sequences of *M*. *cribricollis* were compared with one model insect (*D*. *melanogaster*) and two coleopterans (*T*. *castaneum* and *D*. *ponderosae*). The complete genomes of these three insects have been sequenced and are available in public databases. Orthologous genes gathered together in the same cluster, and among the total number of 11,515 clusters, 9512 were orthologs (at least contained two species) and 4145 were single-copy gene clusters (S8 Table). As a result, we found 63.33% (5201 hits) unigenes were shared among the four insects. And approximately 80% (7007–6347 hits of 8213) unigenes of *M*. *cribricollis* exhibited the most similar identity with protein encoding sequences from *T*. *castaneum* and *D*. *ponderosae*. However, nearly 9.73% (799 hits) of the unigenes of *M*. *cribricollis* had no orthologous proteins with the other three species (Fig 2).

### DEGs analysis

**Fig 3:**
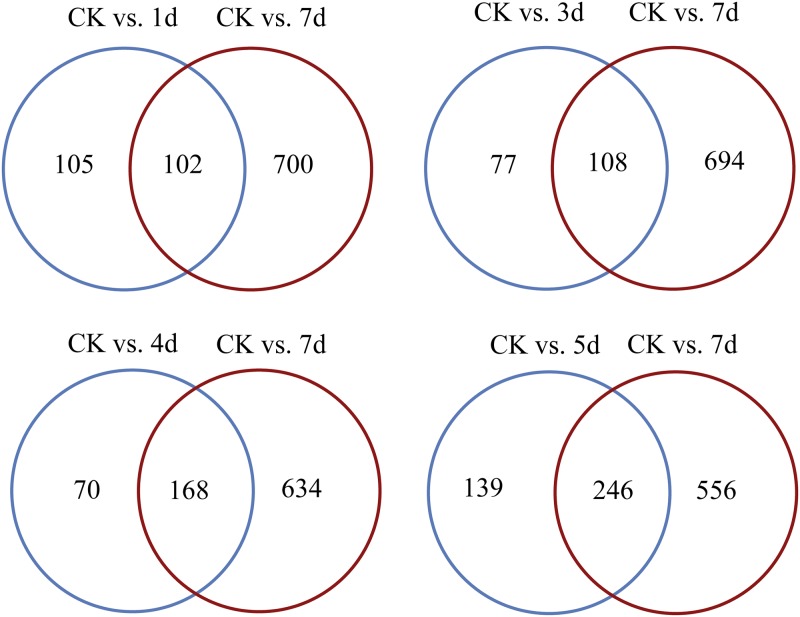
Analysis of differentially expressed genes (DEGs). (A) An overview of DEGs in the control group vs. the five infective libraries. The number of up-/down-regulated genes are marked above each bar, the black bars indicate up-regulated genes, and the blue bars indicate the down-regulated genes. (B) DEGs comparison between CK (control) vs. 7d with four other groups (CK vs. 1d, CK vs. 3d, CK vs. 4d, CK vs. 5d). The black circle indicates CK vs. 7d, and the blue circle indicates CK vs. X d. X represents 1d, 3d, 4d, 5d.

A general rise of the DEGs number (115, 88, 120, 204 and 467) was observed from five different groups, except at 3 d, which showed a slight decline (Fig 3A). Moreover, the higher number of DEGs was consistent with higher mortality in the bioassay (Fig 3A). The Venn diagram demonstrated an obvious growth trend of common as well as specific DGEs number, indicating the difference in gene expression between the early and later stages (7 d) of fungal infection, and more genes of the bamboo wireworm might be induced to express by fungal infection (Fig 3B).

### Pathway enrichment analysis

The pathway enrichment analysis showed that 17, 11, 18, 34 and 78 DGEs from each infected library were mapped to various KEGG pathways, respectively. In total, 24 pathways were enriched (p≤0.05), and most enriched pathways were associated with sugar, amino acid and organic acids metabolism. In this study, we primarily focused on immune-related pathways. “Peroxisome (ko04146)” and “Lysosome (ko04142)” were common pathways among all five infected libraries, but the former was only abundant at 3 d (p = 0.0271) and 5 d (p = 0.0466), and the latter was only enriched at 3 d (p = 0.0459) and 7 d (p = 0.0365). The pathway “ABC transporters” (ko02010), which contributes to defense mechanism was enriched at 1 d (p = 0.0431) and 5 d (p = 0.0284). Moreover, two of the three adversities resistant related pathways, “glutathione metabolism (ko00480)” (p = 0.0492) and “Fc gamma R-mediated phagocytosis (ko04666)” (p = 0.0468), exhibited enrichment at 7 d. Further, more crucial defense-related pathways were also explored, including “drug metabolism-cytochrome P450 (ko00982)”, “metabolism of xenobiotics by cytochrome P450 (ko00980)”, “ubiquitin mediated proteolysis (ko04120)” and “phagosome (ko04145)” (S6 Table).

#### Gene Ontology annotation and enrichment analysis

The DEGs were mapped to three major GO terms (Biological Process, Molecular Function and Cellular Component), with an increased number mapped to the BP GO term, which was consistent with the infection process (S7 Table). Compared to the control group, there were 14, 9, 18, 22 and 27 BP terms enriched significantly in five treatment groups (p≤0.05). Several important immune related BP terms including “positive regulation of melanization defense response (GO: 0035008)” (p = 7.57E-05), “defense response (GO: 0006952)” (p = 0.000148), “regulation of innate immune response” (GO: 0045088) (p = 0.006143), “peptidoglycan catabolic process” (GO: 0009253) (p = 0.009060) and “innate immune response” (GO: 0045087) (p = 0.048106) were mainly enriched at 7 d, “regulation of autophagy (GO: 0010506)” (p = 0.028964) was observed at 4 d, and an important stress defense related BP term “oxidation-reduction process (GO: 0055114)” (p = 0.048710) was enriched at 5 d (S7 Table). Furthermore, other unenriched anti-fungal BP terms were also found, like “cellular response to stimulus (GO: 0051716)”, “salivary gland cell autophagic cell death (GO: 0035071)”, “phagocytosis (GO: 0006909)”, “response to stimulus (GO: 0050896)”, “response to stress (GO: 0006950)”, “antimicrobial humoral response (GO: 0019730)”, “signal transduction (GO: 0007165)”, “ion transport (GO: 0006811)”, etc (S7 Table).

### Quantitative RT-PCR

To validate the reliability of DEGs data generated by the Illumina High-Seq technology, 10 unigenes from *M*. *cribricollis* were randomly selected and their expression levels were quantified by qRT-PCR. The primer sequences designed in these reactions are listed in S10 Table. The results of two methods were concordant; for instance, the unigene, c53119.graph_c0, exhibited a similar trend of gene expression with the highest value at 7 d for both measures (S1 Fig).

### Candidate genes

**Fig 4:**
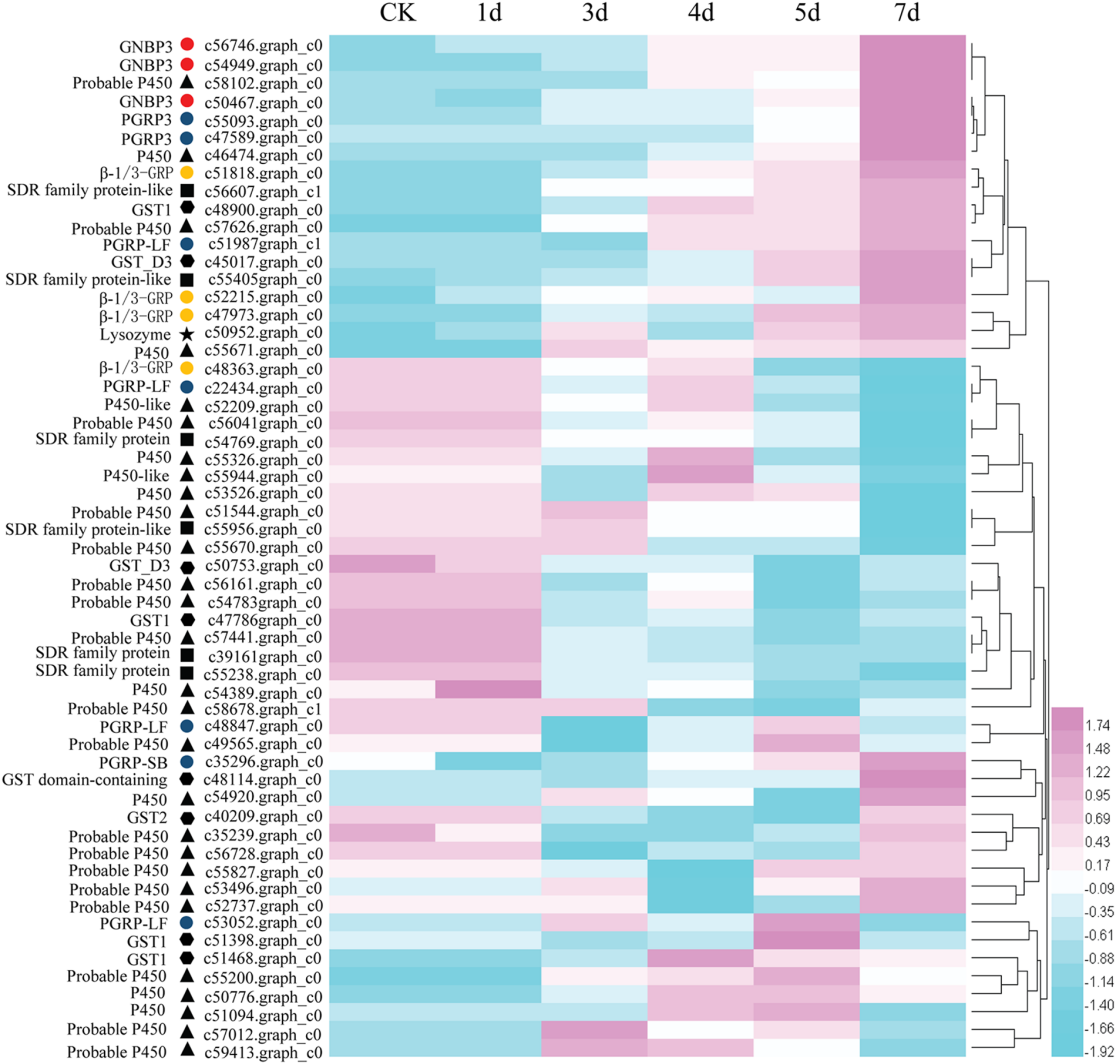
A heat map for the average FPKM values in *M*. *cribricollis* larvae transcriptome over infective process. Each row represents one gene, each column represents one sampling time. Low expression of each cluster is represented in green, intermediate expression is white, high expression is red. Different symbols represent various gene categories. The GNBP designated with a red circle, the PGRP designated with a blue circle, the β-GRP designated with a yellow circle, the lysozyme designated with a black star, the GST designated with a hexagon, the P450 designated with a black trigon, and the SDR designated with a black square.

According to the function description, we found some unigenes were demonstrated to participate in immune defenses or stress responses, including putative unigenes encoding gram negative binding proteins (GNBP), β-1,3-glucan recognition protein (β-GRP), peptidoglycan receptor proteins (PGRP), lysozyme, glutathione S transferase (GST), cytochrome P450 and dehydrogenase/reductase SDR family proteins. Apart from those, many other immune related genes were also identified in the *M*. *cribricollis* larval transcriptomes, like putative unigenes encoding scavenger receptor cysteine-rich domain superfamily protein, Hdd11-like protein, pellino, toll protein, NF-kappa-B, NF-kappa-B inhibitor cactus, nitric oxide synthase, transferrin, ferritin, apoptosis inhibitor, deterin, superoxide dismutase [Cu-Zn]/[Fe], catalase-B, peroxidase-like protein, peroxiredoxin, oxidoreductase, etc (S9 Table). Here, the detail of seven gene categories were described, and the heat-map was produced to compare gene expression levels during different infective time periods. These genes can be mainly classified into three groups depending on the expression patterns, those showing an up-regulation trend, those showing a down-regulation trend and those that fluctuate (Fig 4).

**Fig 5:**
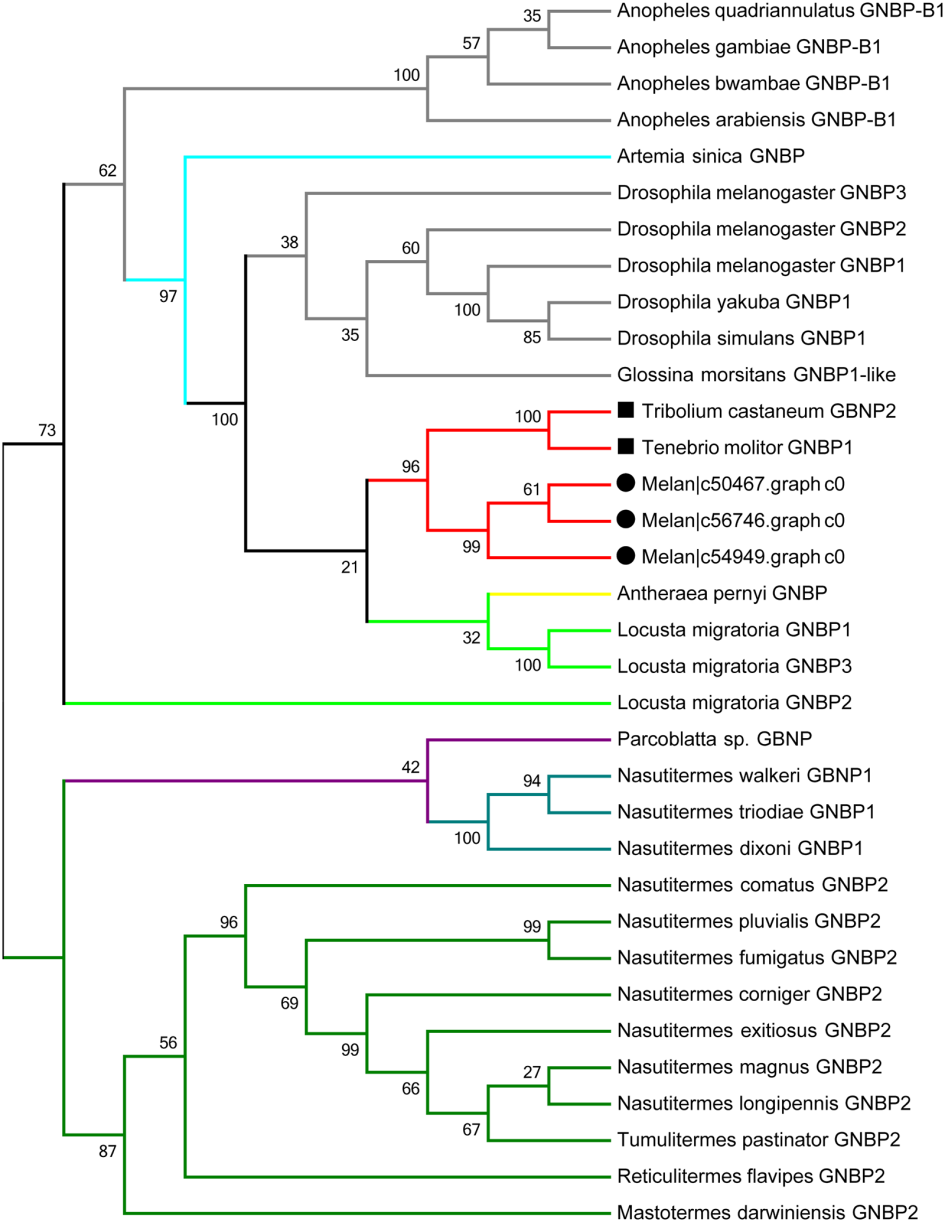
Phylogenetic analysis of GNBPs. Neighbor-joining analysis of GNBP from *M*. *cribricollis* and other insect species using the amino acid sequences of the proteins. Red lines and solid black circles represent sequences from *M*. *cribricollis*. Black hollow circles indicate coleopteran insects. Bootstrap values next to the nodes present the percentage of 1000 replicates supporting the corresponding clade. Other insect species: *Mastoterme darwiniensis*, *Reticulitermes flavipes*, *Tumulitermes pastinator*, *Nasutitermes magnus*, *N*. *comatus*, *N*. *corniger*, *N*. *dixoni*, *N*. *exitiosus*, *N*. *fumigatus*, *N*. *longipennis*, *N*. *pluvialis*, *Na*. *triodiae*, *N*. *walker*, *Parcoblatta sp*., *Anopheles arabiensis*, *A*. *bwambae*, *A*. *gambiae*, *A*. *quadriannulatus*, *Drosophila yakuba*, *D*. *melanogaster*, *D*. *simulans*, *Glossina morsitans*, *Antheraea pernyi*, *Locusta migratoria*, *Tenebrio molitor*, *Tribolium castaneum*, *Artemia sinica*.

Three putative GNBP3 unigenes and four putative β-GRP unigenes were identified, and the expression profile analysis revealed all six unigenes except one (c48363.graph_c0) were up-regulated. The most obvious differences were observed at 7 d post-infection, especially for those two putative GNBP3 unigenes, c56746.graph_c0 (FDR = 0.0016, log2FC = 1.6752) and c54949.graph_c0 (FDR = 0.0012, log2FC = 1.9789), (S9 Table). Phylogenetic analyses were performed to compare three putative GNBP sequences in *M*. *cribricollis* with GNBP homologs from different insects. All GNBP sequences from *M*. *cribricollis* clustered together and with the GNBPs from other Coleopteran insects, which was consistent with the traditional classification (Fig 5). Additionally, seven putative PGRP unigenes were found, including one putative PGRP-SB2 gene, two putative PGRP3 genes and four putative PGRP-LF genes. Among them, four unigenes were DEGs, two putative PGRP-LF unigenes (c22434.graph_c0, c48847.graph_c0) (FDR = 0.0227, log2FC = -1.4676, 7d; FDR = 0.0136/0.0005, log2FC = -1.6555/-2.1492, 3d/4d) were down-regulated, but one putative PGRP-LF unigene (c51987.graph_c1) (FDR = 0.0162, log2FC = 1.5690) and one putative PGRP3 unigene (c55093.graph_c0) (FDR = 0.0158, log2FC = 1.4308) were activated at 7 d compared to the control group. One putative lysozyme unigene (c50952.graph_c0) was identified with substantially higher expression, especially at 7 d (FDR = 3.95E-05, log2FC = 2.0682). In this study, nine putative GSTs unigenes were explored, one was an up-regulated DEG (c51468.graph_c0) (FDR = 0.0388, log2FC = 1.2584, 4d) and two were down-regulated DEGs (c50753.graph_c0, c47786.graph_c0) (FDR = 0.0182, log2FC = -1.5471, 5d; FDR = 0.0363, log2FC = 1.1830, 5d). Additionally, it was found that two putative GSTs unigenes (c48900.graph_c0, c45017.graph_c0) exhibited a gradual increasing trend (P>0.05) according the FPKM mean values. In addition, 34 unigenes encoding P450 and related proteins were annotated, and the majority expressed stably without obvious fluctuation, except two (c56041.graph_c0, c55827.graph_c0) (FDR = 0.0076, log2FC = -1.6617, 7d; FDR = 0.0327/0.0015, log2FC = -1.2659/-1.7223, 3d/4d), which showed significant decline. In contrast, two putative P450 unigenes (c58102.graph_c0, c51094.graph_c0) (FDR = 0.0103/0.0014, log2FC = 1.2985/2.2114, 5d/7d; FDR = 0.0295, log2FC = 1.5251, 4d) displayed a growing trend. Furthermore, six putative SDR unigenes were also analyzed, including two up-regulated DEGs (c55405.graph_c0, c56607.graph_c1) (FDR = 9.23E-05, log2FC = 2.1970, 7d; FDR = 0.0287, log2FC = 1.2136, 7d) and two down-regulated DEGs (c39161.graph_c0, c54769.graph_c0) (FDR = 0.0118/4.49E-07/4.88E-07, log2FC = -1.6476/-2.6543/-2.5685, 4d/5d/7d; FDR = 0.0018, log2FC = -1.5333, 7d) (S9 Table).

## Discussion

Currently, *M*. *cribricollis* larvae are the preeminent threat to sustainable development of bamboo forestry, especially the *Phyllostachys* spp. [4]. *Metarhizium* spp. is a promising entomopathogenic fungi that can be used for the biological control of *M*. *cribricollis*. A comparison analysis of RNA-Seq data between infected and uninfected *M*. *cribricollis* larvae reported here, will advance our understanding of the complexity of insect immune systems in the defense against fungal invasion.

An indoor bioassay was conducted to determine the time point of sample collection. The result suggested that 1 d and 3 d may represent the early phase when fungi started invasion, and 5–7 d could be critical time points likely representing the initiation of the intense interaction between insect and fungi, followed by the moribund stage of bamboo wireworms. Here, the 1 d and 3 d sampling points were chose as representative of the start of infection, 5 d and 7 d were chose as the initiation of fierce interaction, and 4 d was selected as an additional sampling point to make sure there was no omission of critically antagonistic responses for further data mining (Fig 1).

The sequence and *de novo* assembly result suggested that abundant and high quality data was acquired by high-throughput sequencing technology in this study. The high mapped ratios of both *M*. *cribricollis* and CK-Metar indicated the sequencing and assembly data were credible for further analysis. The low ratio of *M*. *pingshaense* WP08 from the infected group was in accordance with the very small percentage of fungi tissue in *M*. *cribricollis* larvae (S1–S4 Tables and Table 1). The functional annotation result showed a low annotation ratio (<50%), it could be that *M*. *cribricollis* is a non-model insect without reference genome information, or genes of this species might possess high mutation rates as well as small peptide-coding genes (S5 Table). Comparative analysis of four insect gene families was performed, most unigenes of *M*. *cribricollis* got hits, only less than 10% had no orthologous sequences, those might encode novel proteins, represent un-translated regions, or correspond to incorrectly assembled transcripts (Fig 2). The reliability of the RNA-Seq data was validated through qRT-PCR, and the result suggested that the RNA-Seq data was reliable for further analysis (S1 Fig).

In this paper, the enriched pathways, GO terms and DEGs, particularly the immune response related ones, were under discussion. With regard to the enriched pathways, the variety and number were both increased gradually, indicating that several pathways were influenced by fungal infection. Some pathways (“lysosome (ko04142)” and “peroxisome (ko04146)”) were ubiquitous, suggesting that these two were likely general regulatory tactics involved in anti-fungi physiological processes. Additionally, some (“Fc gamma R-mediated phagocytosis (ko04666)” and “phagosome (ko04145)”) were specifically enriched at 7 d, which might correspond with the specific process of infection. The discovery of enriched pathways related to sugars, amino acids and organic acids metabolism suggested that fungal infection disturbs the normal growth and development of the insect host (S6 Table). As to GO terms, it was obvious that more GO terms with specific functions were enriched during later infection phase (7 d), especially those related to defense responses (S7 Table). These results indicated that fungal infection activated multiple reactions of the insect host, especially the initiation of more anti-fungi biological processes. This phenomenon corresponded with the larval infection bioassay where larval mortality significantly increased (about 20%) at 7 d, indicating the fierce infective stage of the entomopathogenic fungi. According to the results, it could be found that the majority of DEGs were down-regulated by fungal invasion (Fig 3A), possibly indicating the inhibition effect on wireworms’ healthy growth and development. Meanwhile, the limited sampling time could have potentially influenced the result.

To fully understand the cellular and humoral immune responses of *M*. *cribricollis*, unigenes especially the DGEs, involved in several important immune related BP terms were analyzed. These unigenes were annotated based on orthologous genes from three other insect species, *D*. *melanogaster*, *T*. *castaneum* and *D*. *ponderosae* and comprised of complete gene sequences, fragments, even allelic or splice variants of the same gene. The results revealed some important immune defense or stress response unigenes with different functions; here, we mainly illuminate the putative encoding genes of gram negative binding proteins, β-1,3-glucan recognition protein, peptidoglycan receptor, lysozyme, glutathione S transferase, cytochrome P450, dehydrogenase/reductase SDR family.

Insect innate immune responses are initiated by recognition patterns between pathogen-associated molecular patterns (PAMPs) and pattern recognition receptors (PRRs), consequently activating corresponding signal transduction pathways (e.g. Toll / IMD/JNK, etc.) and finally induce the expression of anti-microbial proteins (AMPs) or other defense molecules [18–22]. Insect hosts have evolved abundant PRRs against a comprehensive spectrum of pathogens, like GNBP, β-GRP, PGRP, etc. GNBPs/β-GRP belong to the same PRR subfamily [23] which binding to β-1,3-glucan or LPS [24–26], and could be constitutively expressed[27] or induced by microbial injection [28–31]. GNBP3 gene could be induced upon septic injury in *D*. *melanogaster* [30] and significantly up-regulated (>2-fold change) in *T*. *castaneum* after being parasite [32]. Experiments have shown that β-GRP gene silencing would increase the mortality of Locusta (*Migratoria manilensis*) and mosquitoe mutants (*Dirofilaria immitis*) [31, 33–34]. Note that in this study six of seven GNBPs/β-GRP unigenes were up-regulated, indicating that PRRs genes were activated by successful fungal infections. PGRPs can bind to peptidoglycans of gram positive/negative bacteria, activate the Toll and IMD signal transduction pathways, immune-related proteolytic cascades and finally phagocytosis [25, 35]. Currently, more than 19 PGRPs genes have been identified [23, 35]. The homologs of PGRP-SA, PGRP-LC and PGRP-SC2 were up-regulated in the pollen beetle (*Meligethes aeneus*) in response to bacterial injection [23]. Six putative PGRP sequences (PGRP1-6) were found in *Galleria mellonella* [35], five PGRPs genes (PGRP-LA, LB, LC, SA and SB) were expressed more conspicuously in *Drosophila* Oregon^R^ adult males with septic injury [36], and several PGRPs (PGRP- SA, SB, LA, LB, LC, LE and LD) were identified in *T*. *castaneum*, with LE and LD not markedly up-regulated after LPS injection [30, 32]. In this study, seven PGRPs genes were also discovered in *M*. *cribricollis* larvae, and classified into PGRP-SB, PGRP-LF and PGRPs. Based on the result, we found that responsiveness of the GNBP3 gene (c56746.graph_c0, c54949.graph_c0), PGRP-LF gene (c22434.graph_c0, c48847.graph_c0, c51987.graph_c1) and *PGRP3* gene (c55093.graph_c0) were the most distinguished, indicating that these encoding PRRs were more sensitive to fungal infection and play key roles in protecting the insect host. Furthermore, obvious differences were noticed at 7 d compared with the control, suggested that *M*. *cribricollis* larvae may start intense resistance at 7 d against *M*. *pingshaense* WP08 infection (S9 Table).

Lysozymes are polypeptides which exhibit antibacterial and antifungal activities [37]). The level and activity of lysozymes are important indices of innate immunity [38]. A lysozyme gene (*HaLyz*) in *Helicoverpa armigera* was reported to be up-regulated after injection of *E*. *coli* or *Beauveria bassiana* but showed different expression patterns [39]. Similarly, in *G*. *mellonella*, lysozyme expression was observed even in naïve larvae revealing its importance in early stage immune response to inhibit *Candida albicans* infection [37]. Larval *Spodoptera* spp. expressed lysozyme at higher levels after *M*. *anisopliae* infection, especially at moribund stage [40]. Interestingly, lysozyme exerted activity in a dose-dependent manner [37,41]; *Paracoccidioides brasiliensis* transition could be inhibited by higher inflammatory concentrations of lysozyme but not lower physiologic concentrations [41]. In this study, one putative lysozyme DEG (c50952.graph_c0) was found, annotated to two BP GO terms; antimicrobial humoral response (GO: 0019730) and salivary gland cell autophagic cell death (GO: 0035071) (S7 Table). The higher gene expression levels in infected larvae compared to the control (S9 Table) indicated that lysozymes might play an important in the *M*. *cribricollis* defense response against *M*. *pingshaense* WP08. However, the mechanisms responsible for fungistatic and/or fungicidal activity of lysozymes are not clear [37]; more work is needed to elucidate their mode of action.

GSTs are major enzymes involved in the detoxification process of endogenous and xenobiotic toxins in multiple organisms [42]. It is a superfamily, and can be generally classified into six major categories, of which Delta and Epsilon are insect-specific subclasses. Insect GSTs work mainly by catalyzing the conjugation of various electrophilic compounds to glutathione (GSH), and also can act as a ligand to capture toxic substances, to achieve detoxification or be involved in resistance development [43–45]. It has been reported that the GST activity was influenced significantly in *Plutella xylostella* larvae infected by *Isaria fumosorosea* [46]. Hou CX (2014) indicated that GSTs could be directly involved in the immune recognition and defense response in silkworms against *B*. *bassiana* [47]. In this study, one putative GST unigene (c50753.graph_c0) was identified from glutathione metabolic process (GO: 0006749), but it was down-regulated. This gene was expressed actively in uninfected larvae but was inhibited after fungal infection, especially on 5d (FDR = 0.0182; log2FC = -1.5471) (S7 and S9 Tables).

Cytochrome P450 (CYPs) are mixed function oxidases and among them cytochrome P450 monooxygenases constitute a large and complex superfamily of heme-thiolate proteins, with representatives in all living organisms [48]. P450s are well known as one of the most rapidly radiating groups of detoxifying enzymes [49] that metabolize toxic substrates via hydroxylation/oxidation [50]. Insect genomes carry hundreds of CYP genes, for instance, about 170 CYP were explored in the mosquito, *Culex quinquefasciatus* [51]. P450 proteins are multi-function enzymes and are involved in insect growth, development, and reproduction, biosynthetic pathways of endogenous compounds, metabolism of insecticides and plant toxic chemicals, detoxification process and defense response against exogenous substance, and other unknown functions [52–53]. Some putative cytochrome P450 related unigenes were identified in the oxidation-reduction process (GO: 0055114) (S7 and S9 Tables), which suggested a potential involvement of P450s in antioxidant response, perhaps in the metabolism of toxic substances produced by *M*. *pingshaense* WP08. However, the stimuli-responsive activity of P450 was not quite obvious in general, only four DEGs (c56041.graph_c0, c55827.graph_c0, c58102.graph_c0, c51094.graph_c0) were up-/down-regulated (S7 and S9 Tables), which may be influenced by the infective stage. Importantly, the oxidation-reduction process in *M*. *cribricollis* larvae was not predominant before 7d.

Dehydrogenases/reductases (SDR) play an important role in various pathogen/biochemical pathways, including intermediary metabolism and biotransformation of xenobiotics, and only 20% of them are well characterized [54–55]. SDRs have been found in animal, plant, nematode and insect [56]. Several putative SDR unigenes with various expression patterns were detected in this study (S9 Table), suggesting their participation in diverse processes in the immune response of this bamboo wireworm against entomopathogenic fungi.

## Conclusion

In conclusion, the immune system of *M*. *cribricollis* larvae against *M*. *pingshaense* WP08 was investigated using the Illumina next generation sequencing technology. Our results indicated that *M*. *cribricollis* larvae could initiate additional defense mechanisms while the fungi could induce serious negative effects that increase with an increase in post-infection time. The fierce infection did not stop until the larvae died. This study is the first to report the genetic background of *M*. *cribricollis* both uninfected and infected by fungi. This result may help the further exploration of insect–fungi interaction mechanisms, and might benefit bio-control strategies combined with molecular biological methods to control this devastating pest.

## Supporting information

S1 Fig RNA-Seq & qRT-PCR Heml analysis. (TIF)

S1 Table All Sample_GC_Q.stat. (DOC)

S2 Table Correlation efficiency. (DOC)

S3 Table *de novo* assembly of *M*. *cribricollis* sequence. (DOC)

S4 Table *de novo* assembly of *M*. *pingshaense* WP08 sequences. (DOC)

S5 Table Summary of annotated unigenes. (XLS)

S6 Table KEGG.stat. (XLS)

S7 Table GO term.stat. (XLS)

S8 Table Orthovenn cluster.stat. (XLS)

S9 Table Interested genes. (XLS)

S10 Table qRT-PCR Primer. (XLS)
