# Supplementary figures and images for: RNA-sequencing analysis of fungi-induced transcripts from the bamboo wireworm Melanotus cribricollis (Coleoptera: Elateridae) larvae
Source: PLoS One. 2018 Jan 16;13(1):e0191187. doi: 10.1371/journal.pone.0191187 (PMC5770045; doi:10.1371/journal.pone.0191187)

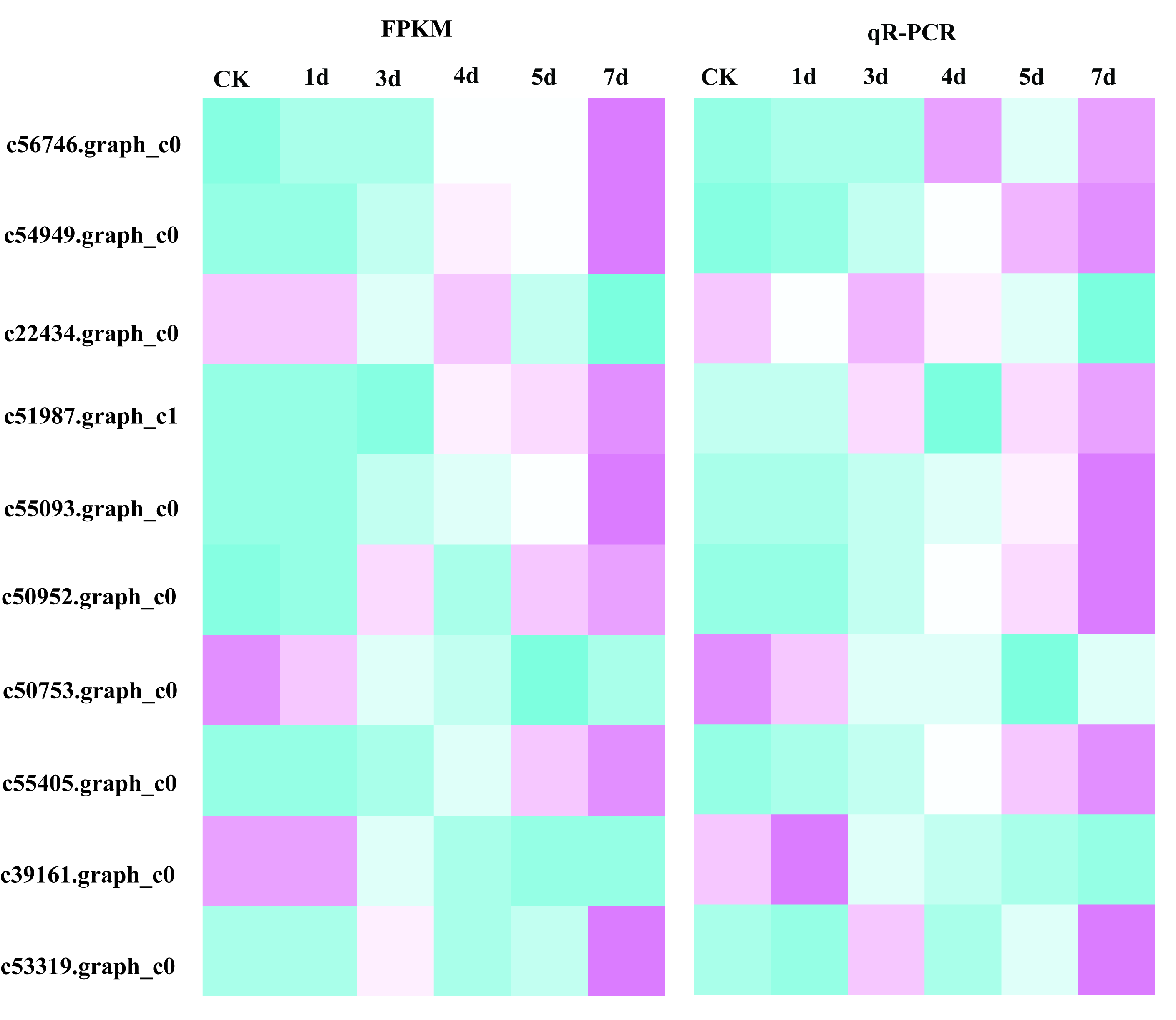

Supplement: S1 Fig — (TIF) [file pone.0191187.s001.tif]
