# Supplementary material for: RNA-sequencing analysis of fungi-induced transcripts from the bamboo wireworm Melanotus cribricollis (Coleoptera: Elateridae) larvae
Source: PLoS One. 2018 Jan 16;13(1):e0191187. doi: 10.1371/journal.pone.0191187 (PMC5770045; doi:10.1371/journal.pone.0191187)
Supplement: S1 Table — (DOC) [file pone.0191187.s002.doc]

S1 Table. All Sample_GC_Q.stat

| **Samples** | **Read Number** | **Base Number** | **GC Content** | **≥Q30 (%)** |
| --- | --- | --- | --- | --- |
| **CK- 1** | 45,615,157 | 9.12E+09 | 41.58% | 95.81% |
| **CK- 2** | 33,453,157 | 6.69E+09 | 41.33% | 96.36% |
| **CK- 3** | 35,423,909 | 7.08E+09 | 40.12% | 96.06% |
| **1d-1** | 51,363,722 | 1.03E+10 | 40.88% | 96.12% |
| **1d-2** | 54,041,984 | 1.08E+10 | 41.29% | 95.80% |
| **1d-3** | 45,251,654 | 9.04E+09 | 41.33% | 95.99% |
| **3d-1** | 43,509,750 | 8.70E+09 | 40.45% | 95.87% |
| **3d-2** | 45,746,034 | 9.14E+09 | 41.44% | 95.44% |
| **3d-3** | 34,340,273 | 6.86E+09 | 40.88% | 95.48% |
| **4d-1** | 42,171,110 | 8.43E+09 | 40.94 | 95.29% |
| **4d-2** | 43,032,384 | 8.6E+09 | 40.76 | 95.62% |
| **4d-3** | 54,546,996 | 1.09E+10 | 40.82 | 95.13% |
| **5d-1** | 43,245,103 | 8.64E+09 | 40.78% | 95.42% |
| **5d-2** | 55,937,266 | 1.12E+10 | 40.80% | 95.38% |
| **5d-3** | 37,282,997 | 7.45E+09 | 40.80% | 95.43% |
| **7d-1** | 34,600,994 | 6.92E+09 | 40.57% | 95.28% |
| **7d-2** | 34,657,463 | 6.93E+09 | 40.97% | 95.73% |
| **7d-3** | 42,450,833 | 8.49E+09 | 40.72% | 95.43% |
| **All** | **776,670,786** | 1.55E+11 | NA | NA |

^NA^ indicates Not applicable.
