# Supplementary material for: RNA-sequencing analysis of fungi-induced transcripts from the bamboo wireworm Melanotus cribricollis (Coleoptera: Elateridae) larvae
Source: PLoS One. 2018 Jan 16;13(1):e0191187. doi: 10.1371/journal.pone.0191187 (PMC5770045; doi:10.1371/journal.pone.0191187)
Supplement: S2 Table — (DOC) [file pone.0191187.s003.doc]

S2 Table. Correlation Efficiency

| **Sample 1** | **Sample 2** | **R^2^** |
| --- | --- | --- |
| **CK-1** | CK-2 | 0.8757 |
| **CK-1** | CK-3 | 0.7750 |
| **CK-2** | CK-3 | 0.7814 |
| **1d-1** | 1d-2 | 0.7771 |
| **1d-1** | 1d-3 | 0.8738 |
| **1d-2** | 1d-3 | 0.7726 |
| **3d-1** | 3d-2 | 0.8709 |
| **3d-1** | 3d-3 | 0.9093 |
| **3d-2** | 3d-3 | 0.8152 |
| **4d-1** | 4d-2 | 0.8986 |
| **4d-1** | 4d-3 | 0.8640 |
| **4d-2** | 4d-3 | 0.8425 |
| **5d-1** | 5d-2 | 0.8366 |
| **5d-1** | 5d-3 | 0.8678 |
| **5d-2** | 5d-3 | 0.8675 |
| **7d-1** | 7d-2 | 0.9668 |
| **7d-1** | 7d-3 | 0.8461 |
| **7d-2** | 7d-3 | 0.8371 |
