# Supplementary material for: RNA-sequencing analysis of fungi-induced transcripts from the bamboo wireworm Melanotus cribricollis (Coleoptera: Elateridae) larvae
Source: PLoS One. 2018 Jan 16;13(1):e0191187. doi: 10.1371/journal.pone.0191187 (PMC5770045; doi:10.1371/journal.pone.0191187)
Supplement: S3 Table — (DOC) [file pone.0191187.s004.doc]

**S3 Table. *de novo* assembly of *M. cribricollis* sequences**

| **Length span** | **Total Number (%)** | | |
| --- | --- | --- | --- |
|  | **Contigs** | **Transcripts** | **Unigenes** |
| **0-300** | 10,683,355 (99.59%) | 35,025 (27.79%) | 27,263 (42.62%) |
| **300-500** | 19,555 (0.18%) | 24,735 (19.63%) | 15,303 (23.92%) |
| **500-1000** | 11,373 (0.11%) | 21,909 (17.38%) | 9050 (14.15%) |
| **1000-2000** | 6974 (0.07%) | 20,696 (16.42%) | 6280 (9.82%) |
| **2000+** | 6057 (0.06%) | 23,665 (18.78%) | 6076 (9.50%) |
| **Total Number** | 10,727,315 | 126,031 | 63,973 |
| **Total Length** | 472,953,984bp | 1.49E+08bp | 49,424,921bp |
| **N50 Length** | 46bp | 2382bp | 1670bp |
| **Mean Length** | 44.09bp | 1182.63bp | 772.59bp |
