# Supplementary material for: RNA-sequencing analysis of fungi-induced transcripts from the bamboo wireworm Melanotus cribricollis (Coleoptera: Elateridae) larvae
Source: PLoS One. 2018 Jan 16;13(1):e0191187. doi: 10.1371/journal.pone.0191187 (PMC5770045; doi:10.1371/journal.pone.0191187)
Supplement: S4 Table — (DOC) [file pone.0191187.s005.doc]

**S4 Table. *de novo* assembly of *M. pingshaense*** WP08 sequences

| **Length span** | **Total Number (%)** | | |
| --- | --- | --- | --- |
|  | **Contigs** | **Transcripts** | **Unigenes** |
| **0-300** | 16,278,107 (99.89%) | 12405 (26.95%) | 11944 (42.49%) |
| **300-500** | 4833 (0.03%) | 4593 (9.98%) | 3834 (13.64%) |
| **500-1000** | 3640 (0.02%) | 5088 (11.05%) | 3179 (11.31%) |
| **1000-2000** | 4055 (0.02%) | 8057 (17.50%) | 3904 (13.89%) |
| **2000+** | 5305 (0.03%) | 15887 (34.51%) | 5246 (18.66%) |
| **Total Number** | 16,295,940 | 46,030 | 28,107 |
| **Total Length** | 613,016,162 | 86,512,165 | 31,199,898 |
| **N50 Length** | 39bp | 3654bp | 2547bp |
| **Mean Length** | 37.62bp | 1879.47bp | 1110.04bp |
